# Exogenous Methyl Jasmonate Improves Cold Tolerance with Parallel Induction of Two Cold-Regulated (*COR*) Genes Expression in *Triticum aestivum* L.

**DOI:** 10.3390/plants10071421

**Published:** 2021-07-12

**Authors:** Natalia Repkina, Anna Ignatenko, Ekaterina Holoptseva, Zbigniew MiszalskI, Paweł Kaszycki, Vera Talanova

**Affiliations:** 1 Institute of Biology of the Karelian Research Centre of the Russian Academy of Sciences, Pushkinskaya St. 11, 185910 Petrozavodsk, Russia; angelina911@ya.ru (A.I.); holoptseva@krc.karelia.ru (E.H.); talanova@krc.karelia.ru (V.T.); 2 W. Szafer Institute of Botany, Polish Academy of Sciences, ul. Lubicz 46, 31512 Kraków, Poland; z.miszalski@botany.pl; 3 Department of Plant Biology and Biotechnology, Faculty of Biotechnology and Horticulture, University of Agriculture in Krakow, al. 29 Listopada 54, 31425 Kraków, Poland; pawel.kaszycki@urk.edu.pl

**Keywords:** phytohormone, tolerance, photosynthesis, antioxidants, gene, wheat

## Abstract

Methyl jasmonate (MJ) is an important plant growth regulator that plays a key role in tolerance to biotic and abiotic stresses. In this research, the effects of exogenous MJ on cold tolerance, photosynthesis, activity and gene expression of antioxidant enzymes, proline accumulation, and expression of cold-regulated (*COR*) genes in wheat seedlings under low temperature (4 °C) were investigated. Exogenous MJ treatment (1 µM) promoted wheat cold tolerance before and during cold exposure. Low temperature significantly decreased photosynthetic parameters, whereas MJ application led to their partial recovery under cold exposure. Hydrogen peroxide (H_2_O_2_) and malondialdehyde (MDA) levels increased in response to low temperature, and this was counteracted by MJ application. Exogenous MJ significantly enhanced the activities of antioxidant enzymes and upregulated the expression of *MnSOD* and *CAT* during cold exposure. MJ application also led to enhanced proline content before 4 °C exposure, whereas the *P5CS* gene expression was upregulated by MJ’s presence at both normal (22 °C) and low (4 °C) temperatures. It was also shown that MJ tended to upregulate the expression of the *COR* genes *WCS19* and *WCS120* genes. We conclude that exogenous MJ can alleviate the negative effect of cold stress thus increasing wheat cold tolerance.

## 1. Introduction

Low temperature is a major environmental stress that limits distribution, growth, development, and yield of crop plants. Plants grown in temperate regions have evolved various mechanisms to tolerate low temperature stress [1]. Plant hormones such as abscisic acid, gibberellins, brassinosteroids, ethylene, salicylic acid and jasmonates are known to play essential roles in the regulation of these processes [2]. 

Jasmonates, such as jasmonic acid (JA) and its methyl ester, methyl jasmonate (MJ), act as signaling molecules to coordinate plant stress responses to biotic and abiotic stresses. They are lipid-derived oxylipins produced as a result of lipoxygenases-mediated oxygenation of polyunsaturated fatty acids [3,4]. These crucial plant hormones are known to modulate morphological, physiological, and biochemical processes in plants, playing important roles in regulation of plant growth and development, flowering, trichome initiation, and leaf senescence [2,5,6]. In particular, they mediate defense responses against herbivores, necrotrophic pathogens, and nematodes as well as mutualistic symbiotic microorganisms [3,7]. Moreover, they regulate adaptation of plants to abiotic stresses including ultraviolet radiation, wounding, drought, salinity, heavy metals, ozone, heat, and cold [2,6,7,8,9,10,11,12,13,14,15].

Recent studies have provided evidence supporting the important role of jasmonates in plant cold tolerance by their involvement in physiological, biochemical, and molecular responses [2]. Cold stress elevated endogenous JA levels in *Arabidopsis* [16], rice [17], and wheat [18]. Exogenous application of MJ significantly enhanced *Arabidopsis* freezing tolerance, while blocking jasmonate biosynthesis and its signaling role led to increased sensitivity to cold [2,16]. Additionally, it was shown that transgenic wheat plants with high *AtOPR3* expression levels were tolerant to short-term freezing [19].

MJ has focused much attention because of its ability to enhance chilling tolerance in the fruits of tropical and subtropical plants like banana, mango, avocado, and papaya at cold storage [7,20,21]. The role of MJ in alleviating the chilling injury of these fruits was attributed to the production of cryo-protective agents, proteinase inhibitors, polyamines, ABA, and antioxidants as well as to its potential of lowering the activity of lipoxygenases [13]. Exogenous application of MJ proved effective in improving chilling tolerance of pea [22], rice [23], and cucumber [24]. 

Low temperature leads to an increased generation of reactive oxygen species (ROS) such as hydroxyl radical (OH^–^^•^), superoxide radical (O_2_^–^^•^), and hydrogen peroxide (H_2_O_2_) [25]. ROS overproduction can cause damage of lipids, proteins, and nucleic acids. Plants possess an antioxidant system (AOS) that helps to mitigate the oxidative stress. This system includes the antioxidant enzymes superoxide dismutase (SOD), peroxidase (POD), catalase (CAT), and ascorbate peroxidase (APX), as well as non-enzymatic antioxidants such as glutathione, ascorbic acid, carotenoids, and proline [26]. The efficacy of the plant antioxidant defense system can be evaluated by assessment of the activities of the antioxidant enzymes and determination of the level of non-enzymatic antioxidants [25]. It was found out that low, non-injuring temperatures could promote the activity of antioxidant enzymes and enhance the level of non-enzymatic antioxidants in plants [1,27]. However, only a few studies have investigated the beneficial roles of MJ on plants under cold-induced antioxidant responses. 

In recent years, significant progress has been made towards a better understanding of the molecular mechanisms of cold and freezing tolerance in plants [28,29,30]. In particular, it was found that the INDUCER of CBF EXPRESSION (ICE) transcription factors positively regulated cold tolerance by activating C-REPEAT BINDING FACTORSs (CBFs). The CBF transcription factors, by binding to the C-repeat (CRT)/dehydration-responsive element, activated cold-regulated (*COR*) genes, leading to enhanced cold (freezing) tolerance [31]. Other studies also showed that JA positively regulated the ICE–CBF signaling pathways to enhance freezing tolerance in *Arabidopsis* [2,16]. Plant exposure to cold rapidly elevated endogenous JA levels by inducing JA biosynthesis genes such as *LOX1*, *AOX1*, *AOC1*, and *JAR* in *Arabidopsis* [16], and *OsLOX2*, *OsAOS*, and *OsAOC* in rice [17]. Furthermore, exogenous application of MJ significantly enhanced the freezing tolerance of *Arabidopsis* seedlings, either with or without cold acclimation [2,16]. On the contrary, blocking JA biosynthesis and the signaling pathway in *Arabidopsis* mutants (lox2, aos, jar, and coi1) increased their sensitivity to freezing stress [16]. However, the detailed role played by jasmonates in mediating freezing responses and the molecular mechanisms of these regulatory processes are not yet fully understood. Moreover, there is only scarce data regarding MJ-regulated cold response in crops. Despite our knowledge on the close interaction between components of JA signaling and cold-regulated transcription factors as well as on JA functioning as a crucial upstream signal to the ICE–CBF/DREB1 pathway, very few reports are available on the molecular mechanisms underlying JA-mediated improved tolerance to low temperature [13].

In the present study, to further elucidate the protective effects of exogenous MJ and its regulatory role under cold stress, we investigated photosynthetic parameters, activities, and gene expression of antioxidant enzymes, proline content, and the expression of genes encoding three COR proteins in cold-stressed wheat seedlings. 

## 2. Materials and Methods

### 2.1. Plant Material, Growth Conditions, and Treatments

Seeds of winter wheat (*Triticum aestivum* L. cv. Moskovskaya 39) were purchased from Tula Research Institute of Agriculture, Russia. The seedlings were grown during 7 days on a Hoagland nutrient solution (pH 6.2–6.4) in the growth chamber at temperature condition of about 22 °C, relative humidity of air approximately 60–70%, photosynthetic photon flux density of 180 µmol m^−2^ s^−1^ and a photoperiod of 14 h. Then, the seedlings were exposed to a hardening temperature of 4 °C for 7 days. One day before the cold exposure, the tested seedlings were incubated in the MJ solution (1 µM) (Sigma-Aldrich, St. Louis, MO, USA), whereas the MJ-untreated plants were used as the control. All other conditions remained unchanged. All measurements were performed on the first leaf of wheat seedlings. The growth measurements were completed with the focus on the primary root length. Dry weight (DW) was evaluated after drying the shoots and roots to a constant weight at 80 °C. 

### 2.2. Determination of Cold Tolerance

The cold tolerance of plants was estimated as the temperature at which 50% palisade mesophyll cells died after cooling leaf portions in a thermoelectrical microrefrigerator TZhR–02/–20 (Interm, Moscow, Russia) for 5 min, with sequential change of temperature at an interval of about 0.4 °C [32]. The viability of palisade cells after cooling was determined on the basis of chloroplast disruption and cytoplasm coagulation, using LOMO Micmed-2 light microscope (LOMO, St. Petersburg, Russia). Cold tolerance was expressed as LT_50_ (Lethal Temperature for 50% palisade mesophyll cells).

### 2.3. Gas Exchange and Chlorophyll a Fluorescence Measurements

Gas exchange parameters including net photosynthetic rate (P_n_), stomatal conductance (G_s_), and transpiration rate (E), were measured on the first fully developed leaves using portable photosynthesis system HCM-1000 (Walz, Effeltrich, Germany). Water-use efficiency (WUE) was calculated as the ratio of net photosynthetic rate to transpiration rate. Chlorophyll *a* fluorescence was measured using a portable chlorophyll fluorometer MINI-PAM (Walz, Effeltrich, Germany). The (F_v_/F_m_) maximum quantum yield of PSII photochemistry was measured after 20-min dark adaptation. 

### 2.4. Determination of H_2_O_2_

H_2_O_2_ content was estimated according to Bellincampi et al. (2000) [33]. The leaf samples (0.05 g) were homogenized in 3 cm^3^ cold acetone and centrifuged at 12,000× *g* for 10 min. Then, supernatant (1 cm^3^) was added to xylenol orange (1 cm^3^). The mixture was incubated for 40 min at room temperature and then centrifuged at 10,000× *g* for 5 min. The absorbance of the mixture was measured at 560 nm against blanks, which were prepared similarly but without plant tissue. The H_2_O_2_ content in the leaves was calculated with an H_2_O_2_ solution-derived standard curve.

### 2.5. Determination of Lipid Peroxidation

The level of lipid peroxidation was assessed by the measurement of malondialdehyde (MDA) content as determined by thiobarbituric acid reaction using the method of Stewart and Bewley (1980) [34]. The wheat leaves (0.1 g) were homogenized with 2 cm^3^ 5% thiobarbituric acid in 20% trichloroacetic acid and centrifuged at 10,000× *g* for 15 min at 4 °C. The mixture was heated at 95 °C in a water bath for 30 min, then cooled quickly in an icebath and centrifuged at 10,000× *g* for 5 min. The absorbance of the supernatant was measured at 532 nm and corrected for non-specific turbidity by subtracting the absorbance at 600 nm. The MDA content was calculated using an extinction coefficient of 155 mM^−1^cm^−1^ and expressed as nmol g^−1^ FW (fresh weight).

### 2.6. Analysis of Antioxidant Enzyme Activities

To determine the activities of antioxidant enzymes, the wheat leaves (0.3 g) were homogenized in 3 cm^3^ of 0.1 M K, Na–phosphate buffer (pH 7.8). The homogenate was centrifuged at 14,000× *g* for 20 min at 4 °C and the supernatant was used for the subsequent enzyme assays. 

SOD activity was assayed based on the ability of the enzyme to inhibit photochemical reduction of nitroblue tetrazolium (NBT) to formazan according to Beauchamp and Fridovich (1971) [35]. The reaction mixture (2 cm^3^) contained 0.1 M K, Na–phosphate buffer (pH 7.8), 9.3 mM methionine, 152.3 µM NBT, 1.1 µM EDTA, 2.4% Triton X-100, and 100 mm^3^ of the enzyme extract. The reaction was initiated by adding of 111.3 µM riboflavin solution (50 mm^3^) and then shaking glass test tubes and placing under light (180 µmol m^−2^ s^−1^) for 30 min. The reaction was stopped by switching off the light. The absorbance was measured at 560 nm. The dark and light control was prepared similarly but without illumination and enzyme, respectively.

CAT activity was determined by decay of H_2_O_2_ (ε = 39.6 M^−1^cm^−1^) per min at 240 nm [36]. The reaction mixture contained 2 cm^3^ of 0.1 M K, Na–phosphate buffer (pH 7.0), 50 mm^3^ of 19.4 mM H_2_O_2_. The reaction was initiated by adding 60 mm^3^ of the enzyme extract.

The assay of peroxidase (POD) activity was based on the increase in optical density caused by the oxidation of guaiacol to tetraguaiacol in the presence of H_2_O_2_ [37]. The reaction medium contained 3 cm^3^ of 0.1 M K, Na-phosphate-buffer (pH 6.2), 30 mm^3^ of 9.5 mM H_2_O_2_, 30 mm^3^ of 9mM guaiacol. The reaction was initiated by adding of 50 mm^3^ of the enzyme extract. The absorbance resulting from the formation of tetraguaiacol was recorded at 470 nm upon one min incubation; the extinction coefficient was 0.0266 µM^−1^ cm^−1^.

Protein content was estimated by the method of Bradford (1976) [38], using bovine serum albumin as a standard. 

### 2.7. Determination of Free Proline Content

Free proline content was determined according to Bates et al. (1973) [39]. The wheat leaves (0.5 g) were homogenized in 10 cm^3^ of 3% sulfosalicylic acid and centrifuged at 5100× *g* for 5 min. Then, 2 cm^3^ of supernatant was mixed with 2 cm^3^ ninhydrin reagent and 2 cm^3^ glacial acetic acid. The mixture-containing test tubes were placed in a boiling-water bath for 1 h and then cooled in an ice bath. The absorbance of the mixture was measured at 520 nm. 

### 2.8. Gene Expression

Frozen leaf tissues were homogenized with liquid nitrogen. Total RNA was extracted using TRizol reagent (Evrogen, Moscow, Russia) as indicated by the manufacturer. Total RNA was treated with RNase-free DNase (Syntol, Moscow, Russia) to remove genomic DNA. The purity of RNA samples and their concentrations were determined spectrophotometrically (SmartSpecPlus, Bio-Rad, Berkeley, CA, USA); the samples with A260/A280 ratios ranging within 1.8–2.0 were used for further analysis. One μg of total RNA was reverse-transcribed using MMLV RT kit (Evrogen, Moscow, Russia) following the supplier’s recommendations. Quantitative real-time PCR was performed using the iCycler iQ Real-time PCR Detection System (Bio-Rad, Berkeley, CA, USA). PCRs were performed using the SYBR Green PCR kit (Evrogen, Moscow, Russia). The PCR conditions were as follows: denaturation at 95 °C for 5 min, followed by 40 cycles of denaturation at 95 °C for 15 s, annealing at 56 °C for 40 s and extension at 72 °C for 45 s. A dissociation curve was generated at the end of each PCR cycle to verify that a single product was amplified using software with the iCycler iQ Real-time PCR Detection System. To minimize sample variations, mRNA expression of the target gene was normalized relative to the expression of the housekeeping gene actin. The mRNA levels of target genes were quantified in comparison to the control by ΔΔCt [40]. The primers were designed (using Primer Design program) for gene-specific transcript amplification (Table S1). Samples of cDNA isolated from plants not exposed to low temperature were taken for reference. Experiments were carried out with the equipment of the Core Facility of the Karelian Research Centre of the Russian Academy of Sciences.

### 2.9. Statistical Analysis

All experiments were repeated three times. The data were subjected to analysis of variance (ANOVA) and to principal component analysis (PCA). The data were processed using Excel 2007 (Microsoft Corp., Redmond, WA, USA) and analyzed with the Statgraphics Plus 5.0 (Statgraphics Technologies, Inc., The Plains, VA, USA) statistical software. Data are presented as mean values ± standard error (SE). The Fisher’s least significant difference (LSD) test was used to compare the treatment means. Differences at *p* ≤ 0.05 were considered statistically significant. 

## 3. Results

### 3.1. Effect of MJ on Growth and Biomass Accumulation of Wheat Seedlings 

**Figure 1:**
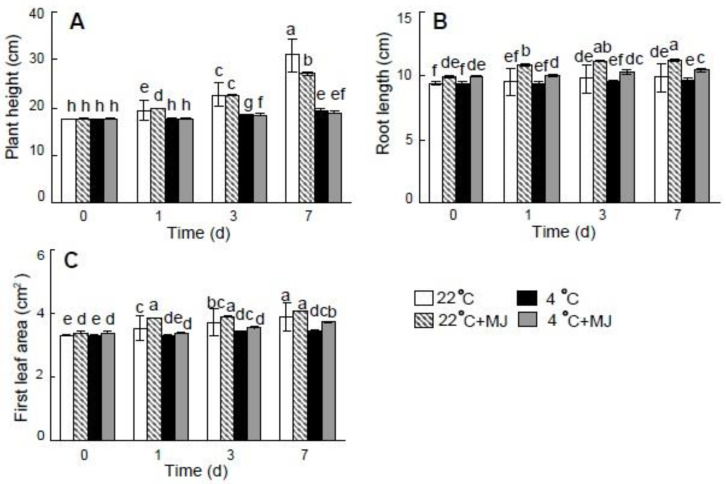
Effect of exogenous MJ application on plant height (**A**) root length (**B**) and first leaf area (**C**). Values represent the mean ± SE (*n* = 90). Different letters indicate significant differences between treatments (*p <* 0.05), determined by Fisher’s least significant difference (LSD) test.

Low temperature (4 °C) adversely affected wheat seedlings growth as compared with the control (22 °C) (Figure 1). The MJ application under 22 °C and 4 °C did not cause an increase in plant height when compared with the temperature impact alone. However, MJ had a positive effect on root length relative to the control (22 °C) and to the variant with low temperature (4 °C) applied alone (Figure 1b).

The pretreatment by MJ alleviated the negative effect of low temperature on the wheat leaf area (Figure 1C). Moreover, MJ led to the elevated biomass accumulation (except for shoot DW) on the seventh day of exposure to 22 °C as compared with the control (Table 1). In addition, the biomass accumulation was greater under the low temperature (4 °C) in the presence of MJ, when compared with the effect of low temperature (4 °C) alone.

### 3.2. Exogenous MJ Improves Cold Tolerance of Wheat Seedlings 

It was found that cold tolerance of wheat seedlings increased after 1 d exposure to low temperature (4 °C). This continued growing and reached a maximum on the seventh day (Table 2). The pretreatment of MJ (1 µM) induced an increase in cold tolerance during the whole period of low temperature treatment (Table 2). Although the observed effect is not considerable, it is still statistically significant.

Table 2 shows the temperature at which 50% palisade mesophyll cells died. The cold tolerance was expressed as LT_50_,°C (Lethal Temperature of 50% palisade mesophyll cells). 

### 3.3. Exogenous MJ Stabilizes Wheat Photosynthesis

**Figure 2:**
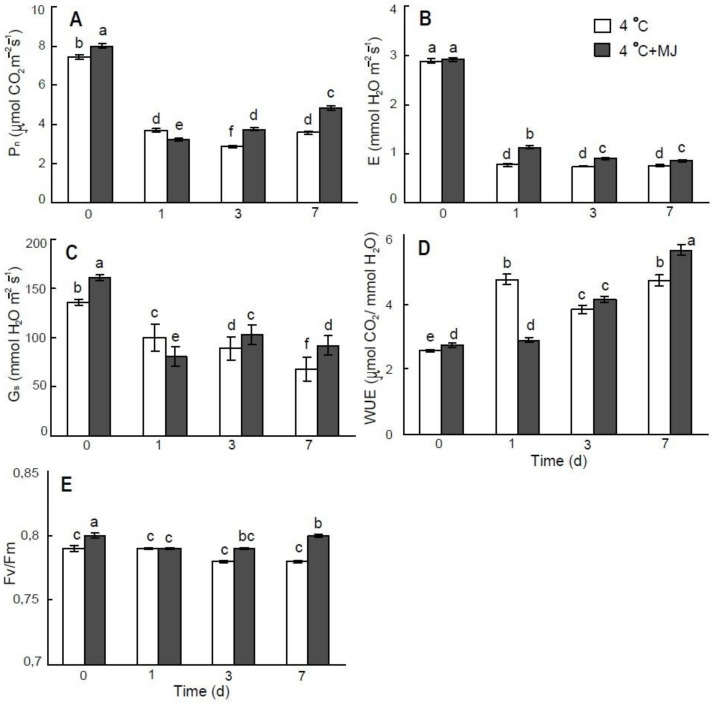
Effect of exogenous MJ application on (**A**) net photosynthetic rate (Pn), (**B**) transpiration rate (E), (**C**) stomatal conductance (Gs), (**D**) water-use efficiency (WUE), and (**E**) maximum efficiency of PSII photochemistry (Fv/Fm) of wheat plants exposed to low hardening temperature of 4 °C. Values represent the mean ± SE (*n* = 15). Different letters indicate significant differences between treatments (*p <* 0.05), determined by Fisher’s least significant difference (LSD) test.

The net photosynthetic rate (P_n_), stomatal conductance (G_s_), and transpiration (E) of wheat seedlings decreased significantly on day 7 under low temperature conditions (to 48%, 27%, and 50% of the initial level, respectively) (Figure 2). However, exogenous MJ application slightly reduced these negative effects in cold-treated plants to 60%, 29%, and 56%, respectively. Water-use efficiency (WUE) was elevated immediately after cold exposure of the wheat seedlings (Figure 2D, white bars). The MJ treatment improved this parameter; however, the effect was delayed and observed on day 7 of the test (Figure 2D, grey bars). Maximum quantum yield of PSII photochemistry during cold treatment remained on the initial level. Exogenous MJ application slightly improved Fv/Fm under low temperature conditions (Figure 2E). 

### 3.4. Impact of Exogenous MJ on the H_2_O_2_ and MDA Content in Wheat 

The estimation of H_2_O_2_ content in wheat leaves showed a slight increase within the first day of exposure to 4 °C, followed by a further decrease during days 3–7 (Table 3). The H_2_O_2_ content in leaves in the presence of MJ did not change throughout the low temperature treatment experiment (Table 3). The MDA content in leaves increased during the first day of the cold treatment and remained at the same level throughout the 7 days. Pretreatment with MJ led to a slight increase in the MDA content (Table 3).

### 3.5. Exogenous MJ Enhances Activity of Antioxidant Enzymes and Expression of Their Genes in Wheat Leaves

**Figure 3:**
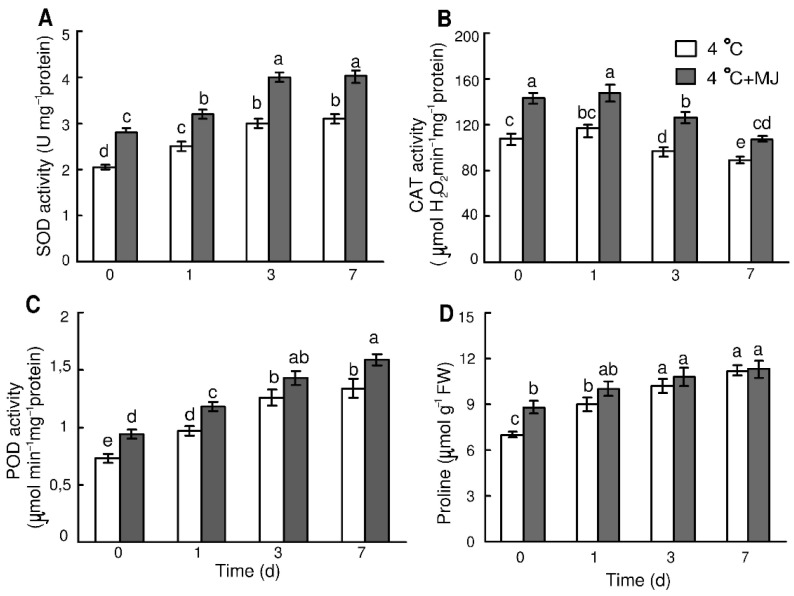
Effect of exogenous MJ application on activity of (**A**) SOD and (**B**) CAT, (**C**) POD, and (**D**) proline content in the leaves of wheat plants exposed to low hardening temperature of 4 °C. Values represent the mean ± SE (*n* = 15). Different letters indicate significant differences between treatments (*p <* 0.05), determined by Fisher’s least significant difference (LSD) test.

A temperature of 4 °C induced an increase in SOD activity in wheat leaves during the whole cold period (Figure 3A). On day 7, the enzyme activity was higher than the initial level by approximately 53%. Exogenous MJ application induced an increase in SOD activity before cold exposure and then it caused additional enhancement of its activity during the whole cold period (on day 7 the SOD activity was higher than the initial level by 97%).

**Figure 4:**
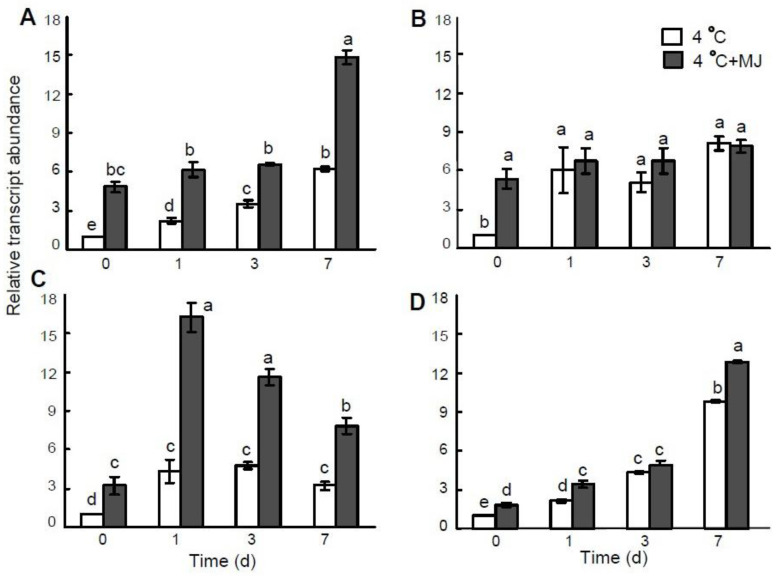
Effect of exogenous MJ application on gene transcription of (**A**) *MnSOD*, (**B**) *FeSOD*, (**C**) *CAT*, and (**D**) *P5CS* in the leaves of wheat plants exposed to low hardening temperature of 4 °C. Values represent the mean ± SE (*n* = 9). Different letters indicate significant differences between treatments (*p <* 0.05), determined by Fisher’s least significant difference (LSD) test.

In a parallel assessment, the accumulation of *MnSOD* and *FeSOD* gene transcripts was established in wheat plants exposed to cold hardening (Figure 4A,B). The mRNA content of the *MnSOD* gene in leaves grew upon the first day of exposure, and within 7 daysit reached a maximum (Figure 4A). Exogenous MJ treatment resulted in a significant increase in the mRNA level of the *MnSOD* gene before cold treatment. Upon prolonged cold exposure, the transcription of this gene was additionally elevated within 3–7 days. The mRNA level ofthe *FeSOD* gene also increased during day 1 of low temperature exposure, and on day 7, the mRNA transcription level of this gene was the highest (Figure 4B). However, the MJ application significantly heightened the mRNA level of the *FeSOD* gene only before cold exposure. 

CAT activity in wheat leaves was slightly elevated at a temperature of 4 °C only within day 1, and then it declined during days 3–7 (Figure 3B). MJ application promoted a significant increase in CAT activity, however, by the end of hardening (day 7); the CAT activities of controls and MJ-treated plants were lower compared to their initial levels. 

The transcription level of the *CAT* gene increased through days 1–3 of exposure to 4 °C (Figure 4C). By the end of the experiment (day 7), the content of the *CAT* gene transcripts decreased but was still higher than the initial level. Exogenous MJ promoted the *CAT* gene transcription before cold treatment. Subsequent MJ application significantly stimulated the expression of this gene at a temperature of 4 °C during the first day, then the mRNA level decreased but remained considerably higher compared to the MJ-untreated plants (Figure 4C).

The POD activity in wheat leaves became elevated by approximately 33% compared to the initial level during the first day after exposure to cold, and continued growing within the 7 days of the test (Figure 3C). MJ application promoted the POD activity by approximately 29% as compared to the initial level before the cold exposure. At the temperature of 4 °C, the MJ application induced additional enhancement of POD activity throughout the exposure (Figure 3C).

**Figure 5:**
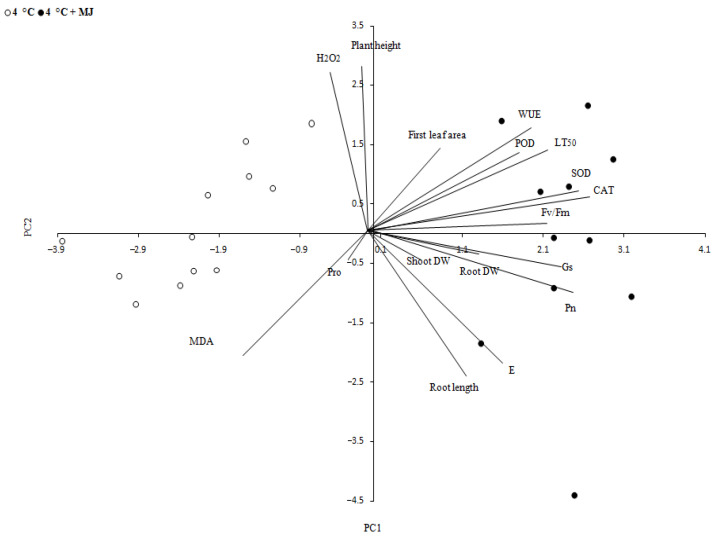
Principal component analysis (PCA). A correlation circle showing a projection of original variables (lines) in the principal components space PC1 and PC2. The lines closeness indicates how much correlation exists between the variables. Graphical representations are obtained from evaluation of parameters studied under a low temperature of 4 °C and 4 °C +MJ (exposure time: 168 h).

The PCA data also support the involvement of MJ in antioxidant enzyme activity under low temperature influence (Figure 5). The PCA analysis was performed to display the maximum amount of variation in a data profile within a few principal components and to understand relations between variables. As could be expected, physiologically related parameters and processes tended to display correlated output values. The analysis of some parameters considering two types of treatment (4 °C and 4 °C + MJ) showed two components, PC1 accounting for 34.4% and PC2 representing 56.7% of total variance.

### 3.6. Exogenous MJ Enhances Free Proline Content 

The low temperature treatment caused an increase in proline content in the leaves (Figure 3D). In particular, proline content grew within the first day of exposure to 4 °C and reached a maximum on day 7. The MJ-treated wheat plants had an enhanced proline content during the initial period (day 1) of cold exposure compared to the untreated seedlings (Figure 3D).

Proline accumulation in wheat leaves correlated with the rise in the *P5CS* gene transcript level, encoding enzyme Δ’-pyrroline-5-carboxylate synthase (P5CS) (Figure 4D). MJ application resulted in a significantly higher mRNA content of this gene before cold exposure and on the first and the seventh days of cold stress. 

### 3.7. Effect of Exogenous MJ on COR Genes Expression in Wheat

**Figure 6:**
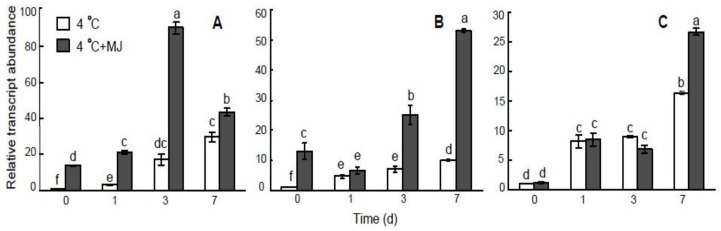
Effect of exogenous MJ application on gene transcription of (**A**) *WCS19*, (**B**) *WCS120*, and (**C**) *WCOR15* in the leaves of wheat plants exposed to low hardening temperature of 4 °C. Values represent the mean ± SE (*n* = 9). Different letters indicate significant differences between treatments (*p <* 0.05), determined by Fisher’s least significant difference (LSD) test.

The results shown in Figure 6 demonstrate the expression of the *COR* genes, including *WCS19*, *WCS120*, and *WCOR15*, under cold stress and upon exogenous MJ application. The MJ-inducible expression of *WCS19* and *WCS120* genes was detected before cold exposure (Figure 6A,B), whereas MJ did not affect the expression of the *WCOR15* gene. At low temperature conditions (4 °C), *WCS19*, *WCS120*, and *WCOR15* transcripts showed high levels of accumulation in wheat leaves. Interestingly, cold-induced expression levels of *WCS19*, *WCS120*, and *WCOR15* genes were significantly upregulated by exogenous MJ. However, the higher expression of *WCS120* and *WCOR15* were delayed (on days 3 and 7, respectively).

## 4. Discussion

Jasmonates, including MJ, have been shown to affect plants and, among other factors, to enhance stress tolerance; however, they has also proved to have a negative effect on photosynthesis and plant growth [5,9,41]. It is generally known that low temperature limits the photosynthetic activity, chloroplastic electron transport and carbon fixation [42] as well as growth [1]. In the present study, we attempted to describe, both on the molecular and physiological levels, wheat reaction to exogenously applied MJ upon exposure to cold-stress conditions. First, based on the results of Table 1 and Figure 1, we proved that the plant material was significantly affected by low temperature (4 °C) treatment. All the data presented thereafter were focused on the MJ effect on plants exposed to cold stress, while the cold-stressed seedlings not treated with MJ served as controls.

It was found that the applied low temperature affected growth of the wheat seedlings; however, the MJ pretreatment slightly enhanced the root dry weight, as well as the dry weight of the shoot on the last (seventh) day of the experiment. MJ also led to an increase in root length under optimal and low temperatures, whereas it did not affect the height of wheat seedlings. Some other authors demonstrated the divergent (either positive, or negative) effects of MJ on growth parameters of various plants that were often revealed in a dose-dependent manner, and were observed under optimal as well as under stress conditions [43]. It should be noted here that in wheat, similarly to our findings, root growth stimulation by MJ under optimal conditions was also documented by Avalbaev et al., 2016 and Allagulova et al., 2020 [44,45]. We suggest that the above phenomena may be caused by the role of MJ acting along with the other plant growth regulators and affecting various plant developmental processes such as the cell cycle, root formation, vegetative growth, etc.

In addition, at the low temperature, an increase in leaf area was observed as compared to untreated plants. Consequently, exogenous MJ applied at low concentration mitigated the growth inhibition caused by chilling. Low temperature may hamper plant growth despite the fact that considerable rates of photosynthetic activity can be maintained. It also results in sink limitation and enables plants to accumulate carbohydrates that act as compatible solutes [46]. Therefore, the reduced growth at low temperature is not only a negative consequence of a hampered metabolism, but might represent a purposive process important for plant survival [47].

In our study, a marked decrease in P_n_ was observed upon cultivation of wheat seedlings under low temperature. These conditions also resulted in a significant reduction in stomatal conductance and transpiration rates. Consequently, the plants exhibited an increase in leaf WUE relative to initial levels. Our results also indicated that application of MJ at low concentration (1 µM) upon prolonged incubation (7 day) further reduced the water loss in leaves of wheat seedlings exposed to low temperature.

Maximum quantum yield of PSII photochemistry (F_v_/F_m_) is widely used as a diagnostic parameter to monitor the adaptation of PSII to low temperatures in cold-tolerant species [46]. In the present study, exogenous MJ application increased F_v_/F_m_ in wheat seedlings exposed to low temperature, which is a result similar to the earlier reported data on exogenous JA administration [15]. It should be noted that the recent investigation on the role of jasmonates in photosynthetic control revealed the occurrence of parallel changes in maximum quantum yield of PSII photochemistry with the expression of genes involved in photosynthesis [48].

In this study, MJ was also shown to counteract the chilling stress and enhance cold tolerance within 3–7 days of MJ application by activation of the antioxidant system and scavenging of reactive oxygen species (ROS). Besides, the above results suggest that the observed upregulation of antioxidant enzyme activities is very likely due to the risen transcripts levels. Our data prove that the content of H_2_O_2_ reached its maximum on day 1 and decreased later from days 3 to 7 during cold hardening. Compared to the control, until day 3 of incubation, the application of MJ decreased the H_2_O_2_ level observed for the temperature of 4 °C. Similarly, pretreatment with MJ of cucumber seedlings repressed the increase in H_2_O_2_ level in leaves caused by chilling stress (15/8 °C) thereby enhancing the chilling tolerance [24].

Several authors have reported that ROS alter the function of a variety of biomolecules (lipids, proteins, nucleic acids) together with lipid peroxidation leading to the loss of membrane integrity [25,49]. In our study, lipid peroxidation was evaluated by malondialdehyde (MDA) level, which was increased only at the beginning of cold hardening as compared to the initial value, while the MJ application repressed its increase under chilling stress conditions. These results suggest that MJ might protect plants by regulating the amount of H_2_O_2_ and MDA under cold stress. Therefore, the treatment with MJ protects wheat plants from oxidative damage and thereby enhances their cold tolerance.

An important way of plants defending against oxidative stress is the induction of activity or gene expression of antioxidant enzymes, such as SOD, CAT, and POD. Higher activity of the enzymatic antioxidant system led to the reduction of ROS level, therefore improving membrane integrity and inducing resistance towards abiotic stresses including cold [25,50]. In our study, the activities of SOD, CAT, and POD in wheat leaves increased after MJ pretreatment of the plants subjected to hardening temperature, as compared to the control. These results are in agreement with that of Ma et al. (2014) [51], who showed that MJ tended to enhance SOD, CAT, and POD activities in wheat under drought stress. 

As shown in the present study, the expression levels of the *MnSOD*, *FeSOD*, and *CAT* genes encoding corresponding enzymes were markedly upregulated in the seedlings under cold hardening temperatures and exposed to MJ pretreatment. For the case of the *FeSOD* gene, its transcript level did not significantly change in wheat treated with MJ. Therefore, the activities of wheat antioxidant enzymes were positively correlated with the expression of their relevant genes, except for the *FeSOD* gene, similarly to the observations of Hernández et al. (2000) [52]. Taken together, these results indicate that exogenous MJ is able to effectively induce ROS scavenging potential by enhancing the activities of antioxidant enzymes and upregulating expression of the respective genes.

Non-enzymatic antioxidant proline is synthesized from glutamate in a process catalyzed by two enzymes, Δ-pyrroline-5-carboxylate synthase (P5CS) and pyrroline-5-carboxylate reductase (P5CR). Proline accumulation in plants subjected to drought, salinity, or freezing is thought to be regulated mainly by P5CS [53,54]. Cold stress-induced proline accumulation and upregulation of P5CS encoding gene has been reported in many plant species, supporting the proposed mechanism that proline may act as an osmoprotectant [1]. Proline also seems to be able to stabilize membranes, to scavenge free radicals, and serve as a source of carbon and nitrogen [53,55].

In our study, low temperature caused significant proline accumulation in wheat leaves. Exogenous application of MJ showed an increase in proline content before cold treatment. The *P5CS* transcript levels were shown to correlate with proline accumulation during cold stress. These data suggest that proline level was regulated at the level of transcription of the gene encoding P5CS enzyme during cold stress and MJ application. Therefore, we expect that the cold stress-induced *P5CS* gene expression in wheat is controlled, at least partly, by the MJ-dependent pathway.

Recently, Hu et al. (2017) [2] suggested that application of JA modulated cold tolerance by regulating CBF/DREB1 transcriptional pathway. Under cold stress, the increased JA levels triggered COI1-mediated degradation of JAZs repressors, and this released ICEs from repression. ICEs then activated CBFs by binding to the DRE/CRT box promoter sequence element [10,16]. CBF, in turn, by binding to the C-repeat (CRT) element, induced *COR* genes, thus leading to tolerance to cold stress [28]. 

As shown here, we analyzed the expression levels of *COR* genes, including *WCS120*, *WCS19*, and *WCOR15*, in response to MJ application under cold stress. The expression of genes *WCS120*, *WCS19*, and *WCOR15* was upregulated by low temperature. The results show that cold-induced expression levels of *WCS19* and *WCS120* genes were significantly upregulated by MJ before cold exposure. Also, at low temperature exposure, MJ application led to an increase in *COR* genes expression, but *WCS120* and *WCOR15* transcripts accumulation were delayed (on day 3 and 7, respectively). These findings may indicate that MJ also modulates plant cold acclimation through the CBF/DREB1-dependent pathway. 

The increasing levels of *WCS120* transcripts in the leaves correlated with the acquired cold tolerance of wheat seedlings exposed to a temperature of 4 °C. The *WCS120* gene encodes dehydrin protein WCS120. Interestingly, Kosová et al. (2011) [56] observed a good correlation between WCS120 accumulation and the cold tolerance of wheat. Dehydrins belong to a large family of LEA (late embryogenesis-abundant) proteins. They are highly hydrophilic molecules and protect other proteins or surfaces of biomembranes from substantial water loss. Dehydrins can serve as markers of plant-acquired freezing tolerance levels both at transcriptional and protein abundance levels [56]. In the case of our plant material, the MJ treatment accelerated this process very effectively.

Previously, it was shown that COR/LEA genes *WCS19* and *WCOR15* encode chloroplast-targeted COR proteins WCS19 and WCOR15 [57,58]. The wheat COR protein WCS19 is a chloroplast stroma protein that belongs to group 3LEA proteins. WCS19 is expressed at low temperature only, but its expression results from the integration of cold stress and light [46]. Several studies have demonstrated a sharp correlation between the accumulation of chloroplast-localized COR protein WCS19 and the whole plant frost tolerance [59] as well as with tolerance to photoinhibition [57]. Interestingly, previous results showed that the expression of the WCS19 gene was correlated with PSII excitation pressure [57]. A variation in the redox status within the photosynthetic machinery was shown to control the expression of WCS19 in wheat [60]. These COR/LEA proteins are proposed to protect the photosystem II function under low temperature conditions [46,57]. Therefore, our results suggest that MJ might induce the expression of mostly *WCS19* and *WCS120*, while the *WCOR15* response is delayed and was observed first on day 7. 

Interestingly, previous results have shown that expression of the *WCS19* gene was correlated with PSII excitation pressure [57]. A variation in the redox status within the photosynthetic machinery has been shown to control the expression of *WCS19* in wheat [60]. Some COR/LEA proteins were proposed to protect the photosystem II function under low temperature conditions [46,57]. This would indicate that these changes are not directly connected with the photosynthetic system. In addition to the above, only small changes in the expression of *FeSOD*, usually present in chloroplasts, were noted. Therefore, one can expect that all changes in photosynthetic apparatus are of a secondary character only. Besides, the above results also suggest that the observed enhancement of antioxidant enzyme activities is due to their increased transcript levels. As shown in our study, the expression levels of these enzymes affect the activities of antioxidant enzymes. Considering the well-known role of jasmonate in plants’ tolerance to biotic stresses together with our data, we can suggest that exogenous MJ application may be used for promoting plant tolerance against different stresses. This may be of particular interest from a practical point of view. We believe that the data presented in this study contributes to a better understanding of the role and mechanisms through which methyl jasmonate reveals its potent action. At the same time, the obtained results bring challenging opportunities for further studies on MJ participation in the development of resistance against low temperature as well as various other physiological stressors.

## 5. Conclusions 

Taken together, the results of our study suggest that exogenous MJ can alleviate the negative effect of cold stress, which enables plants to increase their cold tolerance. This is achieved mainly due to increased expression of the dehydrin proteins *WCS19* and *WCS120*, whereas the observed changes in photosynthetic parameters and antioxidant factors are of a secondary character. Therefore, our results suggest that MJ-induced expression of the wheat *WCS19* and *WCS120* genes as well as delayed upregulation of *WCOR15*, along with the accumulation of the corresponding COR proteins, may be involved in maintaining the activity of the photosynthetic apparatus through regulation of the redox state in chloroplasts under low temperatures.

## Figures and Tables

**Table 1:** Effect of exogenous MJ (1 µM) application on the dry weight (DW) of wheat plants after 7 days of exposure to optimal (22 °C) and low hardening (4 °C) temperature.

Exposure	Shoot DW (mg)	Root DW (mg)
22 °C	26.7 ± 0.1 a	7.9 ± 0.3 b
22 °C + MJ	27.7 ± 0.1 a	8.8 ± 0.5 a
4 °C	19.9 ± 0.5 c	6.3 ± 0.1 c
4 °C + MJ	21.4 ± 0.5 b	7.2 ± 0.4 b

Values represent the mean ± SE (*n* = 30). Different letters indicate significant differences between treatments (*p <* 0.05), determined by Fisher’s least significant difference (LSD) test.

**Table 2:** Effect of exogenous MJ (1 μM) application on cold tolerance (LT_50_, °C) of wheat plants exposed to low hardening temperature of 4 °C.

Treatment	Time (d)			
	0	1	3	7
4 °C	−5.6 ± 0.03 g	−6.8 ± 0.09 e	−7.9 ± 0.08 c	−8.6 ± 0.05 b
4 °C + MJ	−5.9 ± 0.06 f	−7.3 ± 0.04 d	−8.4 ± 0.07 b	−9.1 ± 0.10 a

Values represent the mean ± SE (*n* = 18). Different letters indicate significant differences between treatments at *p <* 0.05, determined by Fisher’s least significant difference (LSD) test.

**Table 3:** Effect of exogenous MJ (1 μM) application on H_2_O_2_ and MDA content in leaves of wheat plants exposed to low hardening temperature of 4 °C.

Time (d)	H_2_O_2_ Content (μmol g^−1^ FW)		MDA Content (nmol g^−1^ FW)	
	4 °C	4 °C + MJ	4 °C	4 °C + MJ
0	56.8 ± 1.6 b	51.6 ± 3.7 bc	23.3 ± 0.4 d	25.7 ± 0.8 c
1	64.3 ± 2.3 a	49.3 ± 1.8 c	31.9 ± 1.4 a	26.6 ± 1.7 b
3	59.5 ± 3.1 ab	54.9 ± 2.6 bc	30.4 ± 1.5 ab	27.7 ± 0.7 b
7	47.8 ± 3.3 c	50.4 ± 3.6 bc	30.3 ± 0.5 a	27.9 ± 0.6 b

Values are means ± SE (*n* = 15). Different letters indicate significant differences between treatments (*p <* 0.05), determinate by Fisher’s least significant difference (LSD) test.

## Data Availability

The authors are confirm that all data, tables and figures in this manuscript are original.

## Supplementary Materials

The following are available online at https://www.mdpi.com/article/10.3390/plants10071421/s1. Table S1. Sequences of primers for real-time PCR analysis.
