# Supplementary material for: Exogenous Methyl Jasmonate Improves Cold Tolerance with Parallel Induction of Two Cold-Regulated (COR) Genes Expression in Triticum aestivum L
Source: Plants (Basel). 2021 Jul 12;10(7):1421. doi: 10.3390/plants10071421 (PMC8309304; doi:10.3390/plants10071421)
Supplement: Supplementary file 1 [file plants-10-01421-s001.zip › plants-1256821-supplementary.pdf]

Table S1. Sequences of primers for real-time PCR analysis.

Gene	Forward and reverse primers	Nucleotide sequence of the primer 5'….3′	NCBI database accession number
MnSOD	forward<br>reverse	ACATAACTGTAACTGCCACG<br>TTGCTCATTTCCCATCTT	AY963808
FeSOD	forward<br>reverse	GGGTCTGGTTGGGTTTG<br>TCGCCTGTCATCCTTGTAATC	JX398977
CAT	forward<br>reverse	TGATACCCAAAGGCACCG<br>GCAGCCAGATAGAACACG	X94352
WCS19	forward<br>reverse	AGGCGACGGAGGAGGCGTGGGAC<br>TTTTCTTTATTGCGTTTGACATT	L13437
WCOR15	forward<br>reverse	GGGAGCAACCTCTTCCATAGTGT<br>CCAACCATCACAACCCTTCACTA	KP266692
WCS120	forward<br>reverse	CACGGCACTGGCGAGAAGAAAGG<br>TGATGTTCTCCATGACGCCCTTC	M93342
P5CS	forward<br>reverse	GGAGACAAGTCCCGTGTTGGTAG<br>GCAGCAACAGCCATTTCACGGAC	KT868850
Actin<br>*(housekeeping gene)*	forward<br>reverse	GGGACCTCACGGATAATCTAATG<br>AACCTCCACTGAGAACAACATTAC	AB181991
